# Effects of Rumen-Protected Glycine and Methionine Supplementation in a Low-Protein Diet on Growth and Development, Slaughter Performance, Meat Quality, and Rumen Microflora of Yudong Black Goats

**DOI:** 10.3390/ani16193002

**Published:** 2026-09-24

**Authors:** Caiye Zhu, Yu Qing, Yue Zhang, Xiaoyan Sun, Hangxing Ren, Youxiang Zeng, Xiaoyu Huang

**Affiliations:** 1 Collage of Animal Science and Technology, Gansu Agricultural University, Lanzhou 730070, China; zhucy@gsau.edu.cn (C.Z.);; 2 Chongqing Academy of Animal Sciences, Chongqing 402460, China; 3 Wulong District Livestock Industry Development Center, Chongqing 408000, China

**Keywords:** Yudong black goat, low-protein diet, rumen-protected amino acid, slaughter performance, serum biochemical index, rumen microbiota

## Abstract

This study aimed to investigate the effects of supplementing rumen-protected glycine and methionine to low-protein diets on the growth and development of Yudong black goats. Sixty 4-month-old Yudong black goats with comparable body weights (21.0 ± 0.74 kg) were randomly assigned to four groups: the control (CON), rumen-protected glycine (GLY), rumen-protected methionine (MET), and mixed-amino-acid (MAG) groups. The CON group was fed a basal diet containing 13.4% crude protein, whereas the GLY, MET and MAG groups received diets with 11.0% crude protein supplemented with 4 g/kg rumen-protected glycine, 4 g/kg rumen-protected methionine, and 2 g/kg rumen-protected glycine + 2 g/kg rumen-protected methionine, respectively. The experimental period lasted 60 days. Compared with the CON group, supplementation with glycine or methionine alone did not affect the growth performance of goats (*p* > 0.05). However, the MAG group exhibited significantly increased longissimus muscle area and C20:3n6 content, together with a significant reduction in ALB (*p* < 0.05). In addition, the relative abundance of *Prevotella* was significantly higher in the MET group than in the CON group (*p* < 0.05), and strong correlations existed between dominant ruminal bacterial genera and indicators of growth and slaughter performance. In conclusion, supplementation with rumen-protected amino acids in low-protein diets can markedly improve slaughter performance and meat quality of Yudong black goats without impairing their growth or normal physiological metabolism.

## Simple Summary

This study explored the feasibility of applying rumen-protected amino acids in low-protein diets for Yudong black goats. A total of 60 four-month-old fattening goats were equally assigned to four groups: a normal crude protein control group and three low-protein diet groups supplemented with rumen-protected glycine, methionine, and their combination, respectively. Following the 60-day feeding trial, supplemental rumen-protected glycine and methionine in low-protein diets did not impair growth performance or normal physiological metabolism in goats. Notably, the mixed amino acid supplementation effectively improved slaughter performance by increasing eye muscle area. Additionally, rumen-protected amino acids regulated the abundance of core rumen microorganisms at the phylum and genus levels. These results indicate that rumen-protected glycine and methionine can optimize low-protein diets for Yudong black goats. This supplementation improves carcass and meat quality while sustaining goat growth performance.

## 1. Introduction

With the upgrading of residents’ consumption and the popularization of the concept of healthy diet, the demand for mutton consumption continues to grow, and the mode of mutton sheep breeding in China has also changed from traditional grazing to standardized captive and semi-grazing [1]. However, the captive breeding of goats has also led to a sharp increase in the demand for a high-quality protein diet. At present, the protein raw materials needed for animal husbandry in China are heavily dependent on imports, which makes the breeding cost high. In addition, it is noteworthy that ruminants exhibit low dietary protein utilization efficiency. Approximately 30%~40% of dietary nitrogen is excreted via feces and urine without being fully utilized, which leads to feed resource waste, soil eutrophication, groundwater contamination, and environmental problems such as excessive nitrate content [2]. The practice of improving animal growth performance by simply increasing the protein content in the diet not only increases the cost of breeding, but also brings additional burden to the environment. The environmental pressure caused by nitrogen emission in ruminant breeding has become a key point restricting the sustainable development of breeding industry [3]. In response to this problem, China’s agricultural development plan in 2023 will incorporate low-protein diet technology into the strategic system.

In recent years, low-protein diets have been widely used in domestic animals breeding. However, excessive reduction in protein levels in diets will inhibit the growth rate of animals and adversely affect their meat quality [4]. In response to this problem, researchers have proposed that nutritional compensation can be achieved by adding functional amino acids, so the ‘low protein + amino acid’ feeding model has attracted much attention in recent years. If untreated amino acids are used directly, most of the protein in the diet will be decomposed and consumed by rumen microorganisms. Therefore, the effective use of amino acids can be ensured by physical or chemical treatment to protect amino acids and make them function at specific sites in the gut [5]. In ruminants, glycine and methionine are key raw materials for protein synthesis, which play an important role in body growth and development, meat quality and immune regulation [6,7,8,9,10].

It has been reported that supplementation of essential amino acids to low-protein diets containing 14% crude protein can improve the dietary protein efficiency of lactating cows without a concomitant decrease in milk protein yield [11]. Rumen-protected methionine supplementation in diets can enhance colonic fermentation and improve production performance of lactating dairy goats [12]. Liu et al. [13] found that reducing the protein level in the diet and feeding rumen-protected sulfur-containing amino acids can improve the carcass quality of Tibetan sheep, and adding rumen-protected methionine can obtain the best edible quality. Zhang et al. [14] supplemented diets with different lysine-to-methionine ratios for Tibetan sheep fed low-crude-protein feed, and found that the treatments did not disrupt jejunal fermentation homeostasis, while effectively improving the nitrogen utilization efficiency and jejunal barrier function of Tibetan sheep. In summary, the ‘low protein + amino acid’ feeding mode has an important impact on the growth and development of livestock. Therefore, studying the relevant regulation mechanism of this model can provide a reference for energy conservation and emission reduction and breeding cost reduction.

However, the combined application of essential and non-essential amino acids in ruminants urgently requires further investigation. Accordingly, we hypothesized that supplementation with rumen-protected methionine and glycine in low-protein diets could improve growth performance and slaughter performance in goats by modulating rumen microorganisms. On this basis, this experiment used 4-month-old Yudong black goats as research subjects to investigate the effects of glycine and methionine supplementation under low-protein conditions on the growth and development, slaughter performance, meat quality, and rumen microorganisms of Yudong black goats, so as to provide a theoretical basis for developing low-cost and high-efficiency nutritional regulation techniques.

## 2. Materials and Methods

### 2.1. Testing Material

Rumen-protected methionine: coating rate was 60%, rumen-protected rate was 86.9%, and small intestine release rate was 82.6%; rumen-protected glycine: coating rate was 50%, rumen-protected rate was 95.8%, and small intestine release rate was 90.2%. All coated amino acids were from Hangzhou Kangdequan Feed Co., Ltd. (Hangzhou, China). The 4-month-old Yudong black goats were provided by the Chongqing Academy of Animal Sciences. This experiment was carried out in a farm in Chongqing.

### 2.2. Experimental Design and Experimental Animals

In this experiment, a total of 60 4-month-old Yudong black goats (36 male goats, 24 female goats) were selected, with an initial average weight of 21 ± 0.74 kg. The experiment lasted for 60 days, including 10 days of pre-experimental period and 50 days of experimental period.

The experimental animals were randomly divided into four treatment groups: the control (CON), glycine (GLY), methionine (MET) and methionine–glycine mixed (MAG) groups. A total of 15 goats were assigned to each treatment, with 3 replicates and 5 goats per replicate. 

The dietary nutrition level and treatment plan were as follows: the control (CON) group was fed a basal diet with a crude protein level of 13.4%; the GLY group was fed a diet with a CP level of 11.0%, and 4 g/kg rumen-protected glycine was added to the diet. The MET group was fed a diet with a CP level of 11.0% and 4 g/kg rumen-protected methionine. The MAG group was fed a diet with a CP level of 11.0%, and 2 g/kg rumen-protected glycine + 2 g/kg rumen-protected methionine was added.

### 2.3. Feeding Management

All goats were raised in open-sided pens, and the pens were fully cleaned and disinfected before the start of the trial. Each goat was equipped with an ear tag and its initial body weight was recorded; standardized vaccination and deworming were performed in accordance with routine procedures. In daily feeding management, goats were fed regularly at 08:00 and 17:00 every day, with sufficient diet and clean drinking water guaranteed, and diet troughs kept sanitized. The health status of the goat herd was observed every day. Pens were disinfected biweekly by alternating 2% sodium hydroxide solution and 0.5% povidone–iodine solution. During the formal trial period, residual diet in each pen was weighed every morning to calculate the average daily dry matter intake (ADFI, g/d) of the previous day, and the daily feeding amount was adjusted to ensure that residual diet accounted for no more than 10% of the total feeding amount.

### 2.4. Diet Formulation

The basal diet was formulated in accordance with the national agricultural standard NY/T 816-2021 [15] Nutrient Requirements of Meat Goats, combined with the actual production conditions of Yudong black goats, targeting an average daily gain of 170 g for growing goats. The crude protein level of the control diet was 13.4%; on the premise of balancing metabolizable energy, the crude protein level of low-protein diets was reduced by 2.4% to 11.0% by adjusting diet ingredients to reduce soybean meal inclusion and modify the proportion of other raw materials, with corresponding rumen-protected amino acids supplemented. All experimental diets consisted of 30% concentrate diet and 70% roughage (air-dried peanut vine roughage). The diet ingredient composition and nutritional levels (dry matter basis) are shown in Table 1.

### 2.5. Collection of Rumen Fluid and Muscle Samples

At the end of the trial, 6 goats were selected from each group for slaughter. After slaughter, the longissimus dorsi muscle between the 12th and 13th ribs was collected, and fascia and fat attached to the muscle were removed. Rumen fluid of each goat was filtered through sterile gauze and divided into three cryogenic tubes, then stored at −80 °C for subsequent testing.

### 2.6. Determination of Indicators

#### 2.6.1. Growth Performance

At the start of the formal trial, all goats were weighed after fasting to record initial body weight (IBW). Live weight was measured every 14 days during the trial, and all goats were weighed on an empty stomach on the morning of the last trial day to record final body weight (FBW). The total diet intake of each pen was recorded daily. Finally, dry matter intake (DMI), average daily gain (ADG) and diet conversion ratio (FCR) of the test goats were calculated in accordance with the formulas recorded in the relevant literature [16].

#### 2.6.2. Slaughter Performance

Goats were fasted for 24 h and deprived of water for 2 h before slaughter. Live weight before slaughter was measured at 07:00 the next morning. After slaughter and a 30 min static placement, carcass weight was weighed, and dressing percentage was calculated. To measure longissimus muscle area, the transverse contour of the muscle between the last two ribs was traced on sulfuric acid paper with a fine-tip marker, and the area was calculated by the formula:

Longissimus muscle area (cm^2^) = muscle height (cm) × muscle width (cm) × 0.70.

#### 2.6.3. Serum Biochemical Index Determination

At the end of the trial, jugular vein blood was collected from 6 goats in each group. Blood samples were loaded into 5 mL anticoagulant tubes, naturally coagulated, centrifuged at 3000 rpm for 15 min, and the upper serum was collected, labeled and temporarily stored at −20 °C. The contents of alanine aminotransferase (ALT), aspartate aminotransferase (AST), total protein (TP), albumin (ALB), glucose (GLU), urea (UREA), creatinine (CREA), total cholesterol (CHOL), triglyceride (TG), high-density lipoprotein-cholesterol (HDL-C), low-density lipoprotein-cholesterol (LDL-C) and non-esterified fatty acid (NEFA) were determined using an automatic biochemical analyzer. Globulin (GLB) was calculated as TP minus ALB; the albumin-to-globulin ratio (A/G) was obtained by dividing ALB by GLB. All operations were performed strictly in accordance with the kit manufacturer’s instructions. Superoxide dismutase (SOD), malondialdehyde (MDA), glutathione peroxidase (GSH-Px), catalase (CAT) and total antioxidant capacity (T-AOC) were assayed following the kit instructions. Growth hormone (GH), gastrin-17 (G-17), insulin-like growth factor-1 (IGF-1), tumor necrosis factor-α (TNF-α), interleukin-4 (IL-4) and interleukin-6 (IL-6) were measured with goat-specific ELISA kits. The absorbance values were read using a microplate reader, and the concentrations of each sample were calculated according to the standard curve.

#### 2.6.4. Determination of Muscle Amino Acid and Fatty Acid Content

The quantification of amino acid and fatty acid profiles of longissimus dorsi muscle samples was entrusted to Qingdao Standard Testing Co., Ltd. (Qingdao, China). Briefly, muscle specimens were vacuum freeze-dried, pulverized and sieved to obtain homogeneous powder subsamples. A portion of the powder was subjected to constant-temperature hydrochloric hydrolysis under nitrogen atmosphere to avoid amino acid oxidation. The hydrolysate was fixed to volume, concentrated, redissolved and membrane-filtered before analysis on a fully automatic amino acid analyzer. Individual amino acid concentrations were determined via ion-exchange chromatography coupled with post-column ninhydrin derivatization and quantified using an external standard method. Owing to its instability under acidic hydrolysis conditions, tryptophan was separately pretreated by alkaline hydrolysis and assayed independently. For free amino acid measurement, muscle powder was extracted with trichloroacetic acid; the supernatant obtained after centrifugation and filtration was loaded onto the instrument. All samples were analyzed in parallel to guarantee data repeatability.

An additional aliquot of lyophilized muscle powder was used for total intramuscular lipid extraction via a chloroform–methanol solvent system. After phase separation, the organic phase was collected, dehydrated and concentrated to yield crude intramuscular fat. The crude lipid underwent sequential saponification and methyl esterification to generate fatty acid methyl esters (FAMEs), which were reconstituted with n-hexane and filtered to prepare test solutions. Gas chromatography equipped with a flame ionization detector (GC-FID) was applied to separate and detect individual fatty acid components. Mixed fatty acid methyl standard mixtures were utilized for peak identification, and the relative proportion of each fatty acid was calculated by the area normalization method. Finally, the total contents of saturated fatty acids (SFAs), monounsaturated fatty acids (MUFAs) and polyunsaturated fatty acids (PUFAs) were summarized, and the n-6/n3 PUFA ratio was calculated for subsequent statistical analysis.

#### 2.6.5. 16S rRNA Gene Sequencing Determination

Rumen fluid was filtered through gauze to remove large feed particles. Rumen microbial DNA was extracted using the TIANamp Stool DNA Kit (TIANGEN, Beijing, China) according to the manufacturer’s instructions. DNA concentration and purity were determined with a NanoDrop 2000 spectrophotometer (Thermo Scientific, Wilmington, DE, USA) and DNA integrity was assessed by agarose gel electrophoresis. The 16S rRNA high-throughput sequencing of rumen fluid samples was completed by Qingdao Standard Testing Co., Ltd. (Qingdao, China). QIIME2 software was used to denoise the original sequencing reads [17]. The DADA2 algorithm was adopted to remove low-quality sequences and chimeras [18]; operational taxonomic units (OTUs) were clustered at 97% sequence similarity. The RDP Classifier algorithm combined with the Silva 138 database (confidence threshold ≥ 70%) was used for taxonomic annotation of species. Seven indices including observed OTUs and the Shannon index were used to evaluate the richness and diversity of microbial communities via α-diversity analysis. Principal coordinate analysis (PCoA) was used to visualize inter-group community differences. Differential genera were screened by LEfSe based on the ASV abundance matrix. The representative sequences corresponding to differential genera were used for functional prediction with PICRUSt2 (v2.4.2). The predicted gene families were annotated against the KEGG Orthology (KO) database, and KEGG pathway and module information was obtained by mapping KO identifiers to the KEGG database. STAMP software (v2.1.3) was applied for statistical testing and visualization of functional-pathway differences.

### 2.7. Data Processing

Statistical analyses were conducted using SPSS 26 software with one-way ANOVA for inter-group comparison. Prior to ANOVA, normality test and homogeneity of variance test were performed to evaluate the prerequisite assumptions for ANOVA. Duncan’s multiple-range test was adopted for post hoc multiple comparisons when the assumptions of ANOVA were met. Results are expressed as mean ± standard error of the mean (SEM). Differences were considered significant at *p* < 0.05, highly significant at *p* < 0.01, and non-significant at *p* > 0.05. A tendency for difference was defined as 0.05 ≤ *p* < 0.10.

## 3. Results

### 3.1. Effects of Supplementing Rumen-Protected Amino Acids to Low-Protein Diet on Growth Performance of Yudong Black Goats

The IBW of goats in all groups was close and maintained at the same level (*p* > 0.05). The ADG ranged from 170.50 g to 177.25 g. Compared with the CON group, supplementation of rumen-protected amino acids to low-protein diets caused no significant changes in DMI and FCR (*p* > 0.05). The above results indicate that regulating dietary protein level and supplementing rumen-protected amino acids can effectively maintain the growth rate of fattening goats (Table 2).

### 3.2. Effects of Supplementing Rumen-Protected Amino Acids to Low-Protein Diet on Slaughter Performance of Yudong Black Goats

The dressing percentage of all fattening goats exceeded 44%, and no significant differences were observed in carcass weight and dressing percentage among all groups (*p* > 0.05). However, the longissimus muscle area of goats in the MAG group was significantly higher than that in the CON group (*p* < 0.05) (Table 3).

### 3.3. Effects of Supplementing Rumen-Protected Amino Acids to Low-Protein Diet on Serum Biochemical Indices of Yudong Black Goats

In terms of serum biochemical indices, the contents of TP and LDL-C in the GLY and MET groups were significantly higher than those in the CON group (*p* < 0.05); the ALB in the MET group was significantly lower than that in the CON group (*p* < 0.05), and CHOL the GLY group was significantly higher than that in the CON group (*p* < 0.05). No significant differences were observed for other indicators. However, compared with the control group, the GLB and A/G showed an increasing trend in the Gly, Met and MAG groups. (0.05 ≤ *p* < 0.10) (Table 4).

### 3.4. Effects of Supplementing Rumen-Protected Amino Acids to Low-Protein Diet on Muscle Amino Acid and Fatty Acid Contents of Yudong Black Goats

No significant differences were observed in TAA, EAA and FAA between the MAG group and the CON group (*p* > 0.05). However, muscle Cystine content in the GLY group was significantly lower than that in the CON group (*p* < 0.05). The relative content of C20:3n6 was extremely significantly higher in the GLY, MET and MAG groups than in the CON group (*p* < 0.001). Isoleucine, Tyrosine and C14:0 tended to be up-regulated in the MAG group relative to the CON group (0.05 ≤ *p* < 0.10) (Table 5).

### 3.5. Analysis of Rumen Microbial Composition

#### 3.5.1. Differential Analysis of Rumen Microbial Community Structure of Goats Fed Low-Protein Diet Supplemented with Rumen-Protected Amino Acids

As shown in Figure 1A, the rarefaction curves of rumen bacterial communities of the four groups flattened out at 40,000 sequencing reads, indicating that the sequencing depth was reasonable. A total of 18,672 OTUs were identified across the four groups, among which 774 OTUs were shared by all groups (Figure 1B). The CON group contained 5303 OTUs, the MET group 4679 OTUs, the GLY group 4447 OTUs, and the MAG group 6565 OTUs. As shown in Figure 1C, samples from the MAG, GLY, MET, and CON groups were intermingled and exhibited extensive overlap. No distinct separation was observed among groups, indicating that the treatments did not significantly alter the overall ruminal microbial community structure.

#### 3.5.2. Alpha Diversity Analysis

Seven alpha diversity indices including Chao1, Shannon, Simpson, Pielou_e, Faith_pd, observed species and Goods coverage were calculated for all four groups (Figure 2). The *p*-values of all indices were greater than 0.05, indicating that different dietary treatments exerted no significant effects on the species richness and diversity of rumen microbial communities. In summary, all treatments had no significant influence on alpha diversity of rumen microbiota (*p* > 0.05).

#### 3.5.3. Effects of Low-Protein Diet Supplemented with Rumen-Protected Amino Acids on Relative Abundance of Goat Rumen Microorganisms

Firmicutes and Actinobacteria were the dominant phyla in all four groups. Firmicutes and Actinobacteria were the dominant phyla in all four groups. At the phylum level, the relative abundance of Bacteroidetes was significantly lower in the treatment groups compared with the CON group (*p* > 0.05) (Table 6). At the genus level, the relative abundance of *Prevotella* was significantly higher in the MET group relative to the CON group (*p* < 0.05). No significant differences were observed in the relative abundance of other genera among all groups (*p* > 0.05) (Table 7).

#### 3.5.4. Correlation Analysis Between Dominant Genera and Growth and Slaughter Performance Indicators

There were certain correlations between dominant rumen genera and growth and slaughter performance indicators. Among them, *Prevotella* and *CF231* were extremely significantly positively correlated with LMA; LW was significantly negatively correlated with *Succiniclasticum*; and DMI and SR were negatively correlated with *CF231* (Figure 3).

#### 3.5.5. Functional Analysis of Differential Genera

Functional prediction of rumen microbial communities for the four treatments was performed based on the KEGG database (Figure 4). Metabolic pathways dominated the functional profiles of rumen microorganisms, including lipid metabolism, glycan biosynthesis and metabolism, metabolism of other amino acids, and energy metabolism, among others. However, none of these predominant pathways exhibited significant differences across the four treatment groups (*p*-adjust > 0.05).

## 4. Discussion

### 4.1. Industrial Application Feasibility of Low-Protein Diet Supplemented with Rumen-Protected Amino Acids

The large-scale development of animal husbandry has boosted the demand for diet raw materials, especially protein raw materials that play a key role in animal growth. China has long faced a supply gap of protein feed resources and relies heavily on international imports. As a core protein feed raw material accounting for about 30% of feed formulations, soybean meals are irreplaceable in livestock diets. It is worth noting that the expansion of intensive breeding has improved production efficiency, but also greatly increased the environmental load brought by livestock waste [19]. With the expansion of breeding density and scale, the discharge of nitrogen pollutants in feces and urine has increased significantly. Therefore, the feed industry needs to strengthen the independent research and development and utilization of non-grain feed raw materials in the future, so as to improve the self-sufficiency of domestic feed resources and guarantee national food security [20]. At present, breeders are developing new feed raw materials on a large scale, and applying food processing by-products such as distillers’ grains, sugar residues and tea seed meal to livestock production. In addition, low-protein feeds have been widely applied in goat breeding [21,22].

The application of low-protein feeds to control animal protein intake can improve the digestibility and utilization efficiency of nitrogen, thereby effectively reducing nitrogen discharge in the breeding process and significantly alleviating adverse impacts on the environment [23]. Based on the law of nitrogen metabolism, adopting the ideal protein pattern in feed formulation and balancing amino acids to reduce excessive protein supply can not only lower breeding costs, but also effectively control nitrogen content in feces and urine [24]. Supplementation with methionine in low-crude-protein diets has no effect on the milk yield, milk fat percentage, milk protein and lactose contents of lactating dairy cows, and can maintain stable whole-body nitrogen balance [25]. Supplementing rumen-protected methionine and lysine to low-protein diets of lactating dairy cows can increase milk yield without changing milk composition [26]. Adding 1% glycine to corn–soybean basal diets can improve the growth performance and feed efficiency of weaned piglets [27]. Cheng et al. [28] found that supplementary glycine or glycine methyl derivative betaine can significantly reduce average backfat thickness and improve lean meat percentage. Won et al. [29] reported that single supplementation of 0.79% glycine in diets can improve growth performance of broilers. The above studies confirm the compensatory effect of amino acid nutrition regulation on livestock production performance. Low-protein feed technology adopts precise nutritional matching and amino acid balance technology to reduce the usage of protein raw materials on the premise of meeting animal growth requirements, which can alleviate the shortage of protein feed resources and significantly reduce the environmental impact caused by nitrogen emission. Although low-protein feed technology has achieved preliminary effects in livestock production, it is far from solving China’s current demand for high-quality protein feed. Future research needs to further promote the matching of ideal protein patterns and amino acid levels, combine low-protein diet models with the development of new feed raw materials, and take into account the nutritional characteristics and anti-nutritional factors of different feed sources.

### 4.2. Effects of Dietary Supplemental Methionine and Glycine on Goat Growth Performance, Slaughter Performance, Serum Biochemical Indices, Meat Quality and Rumen Microorganisms

Animals rely mainly on nutrients from feed during feeding, among which protein is the core nutrient, and amino acids are essential substances for protein synthesis, which play a decisive role in animal growth and development. Reducing dietary crude protein level will inevitably reduce the content of amino acids in feed, leading to insufficient nutrient supply and impaired animal growth performance [30]. Existing studies have pointed out that supplementing rumen-protected amino acids to low-protein feeds can offset the adverse effects caused by insufficient crude protein on animal growth performance [31]. Methionine supplementation to low-crude-protein diets does not alter the milk yield, milk fat percentage, milk protein and lactose concentrations in lactating dairy cows, while sustaining steady nitrogen balance [25]. Supplementation of lysine and methionine at a 1:1 ratio in diets can increase the content of unsaturated fatty acids and improve mutton flavor [32]. Regulating the ratio of lysine to methionine in low-crude-protein diets for Tibetan sheep exerts no adverse effects on the intestinal fermentation homeostasis of Tibetan sheep, while effectively improving the nitrogen utilization efficiency and the jejunal intestinal barrier function [14]. In summary, supplementing amino acids to low-protein diets can alleviate the adverse impacts caused by insufficient crude protein, and improve body nitrogen metabolism and production performance. The present trial further verified the above effects by supplementary rumen-protected methionine and glycine.

Reducing dietary crude protein (CP) is an effective strategy for improving nitrogen-use efficiency and reducing nitrogen losses in ruminant production, but excessive CP reduction may compromise the post-ruminal supply of metabolizable amino acids and consequently limit animal performance. In this experiment, the dietary crude protein level of fattening goats was reduced from 13.4% to 11.0%, with separate or combined supplementation of rumen-protected glycine and methionine. Our findings indicated that ADG and FCR of all treatment groups had no significant differences compared with the CON group, indicating that supplementary rumen-protected glycine and methionine can compensate for the adverse effects of insufficient dietary crude protein on Yudong black goats. Most dietary protein is degraded by ruminal microorganisms. Therefore, the capacity of rumen-protected glycine and rumen-protected methionine to sustain growth under low-crude-protein conditions may reflect improved amino acid balance and metabolic utilization efficiency. Slaughter performance can be evaluated by indicators such as dressing percentage and longissimus muscle area [33]. In this experiment, the dressing percentage of all groups exceeded 44%; the longissimus muscle area of the MAG group was significantly higher than that of the CON group. This indicates that supplementary rumen-protected glycine and methionine can make up for the loss of slaughter performance caused by low dietary crude protein. Studies on pigs also showed that glycine can increase longissimus muscle area to a certain extent without significant difference, and the significant improvement in the MAG group may be due to the synergistic effect between glycine and methionine, which is related to the mixing ratio of the two amino acids [34]. Collectively, the results reflect the complementary utilization effect of the two amino acids. However, gradient supplementation trials were not conducted in the present study; therefore, the precise mechanisms and optimal supplementation ratio of the two amino acids remain to be further investigated. Glycine and methionine are primarily absorbed and utilized in the small intestine of goats. Serum biochemical indices can reflect the nutrient metabolism, health status and internal and external balance of animals [35]. Triglycerides, LDL-C, HDL-C and total cholesterol are important indicators for evaluating body lipid metabolism. Serum biochemical responses further suggest that the low-CP diets supplemented with rumen-protected amino acids altered protein and lipid metabolism without causing broad metabolic dysfunction.

Mutton is rich in amino acids and fatty acids, whose contents determine the nutritional value and umami taste of mutton. The higher the proportion of essential amino acids in total amino acids, the better the nutritional value of meat [36,37]. In the present study, no significant differences were observed in TAA, EAA and FAA between the MAG group and the CON group. Nevertheless, simultaneous supplementation of glycine and methionine showed the potential to increase the concentrations of isoleucine, tyrosine and C14:0. These findings indicate that reducing dietary crude protein level combined with glycine and methionine supplementation could modulate partial amino acid and fatty acid deposition in muscle without compromising the overall nutritional-related amino acid profile of mutton.

The rumen is a vital digestive and metabolic organ of ruminants, and the abundance and diversity of rumen microbial flora can directly reflect the rumen microecological balance and fermentation metabolism status, which exert important effects on host nutrient digestion and absorption, growth and development and immune function [38,39]. Multiple studies have shown that rumen microbial diversity is related to animal breed, age and dietary composition [40]. Different concentrations of cysteamine were supplemented into lamb diets, and study found that the Shannon index differed among various treatment groups [41]. Dietary astragalus polysaccharides supplementation optimizes the rumen microbial community of yaks [42]. The above studies indicate that changes in dietary formula can alter the richness of rumen microorganisms. The alpha diversity results of this experiment showed that there were no significant differences between low-protein treatment groups and the CON group. This suggests that the dietary intervention did not cause a major disruption of overall microbial richness or evenness. Such stability is biologically plausible because the diets were formulated from broadly similar feed ingredients, and moderate changes in CP do not necessarily produce large shifts in the overall rumen microbial community.

Firmicutes and Bacteroidetes are the dominant phyla in ruminant rumen, and play important roles in degrading fibrous and non-fibrous substances [43,44]. The present study found that the relative abundance of most bacterial phyla did not differ significantly across groups. Nevertheless, Bacteroidetes were significantly less abundant in the low-protein treatment groups. As core ruminal bacteria responsible for degrading protein, peptides and non-fibrous carbohydrates, members of Bacteroidetes are highly dependent on nitrogen substrates for growth. The decreased relative abundance of Bacteroidetes under low-protein conditions indicated that insufficient dietary nitrogen supply restricted the proliferation of this phylum. *Prevotella* is one of the dominant ruminal bacterial genera and is involved in the utilization of protein, peptides and starch-derived substrates [45]. The increased abundance of *Prevotella* in the MET group may indicate that methionine supplementation modified the availability of nitrogenous substrates or carbohydrate fermentation products, thereby favoring bacteria capable of utilizing these substrates. Previous studies have demonstrated that rumen-protected methionine can alter rumen fermentation, microbial populations, nitrogen utilization, and intestinal development in goats. However, the magnitude and direction of these effects vary depending on methionine source, dosage, basal diet, and the physiological stage of animals [46]. Similarly, low-CP diets can alter rumen microbial composition while simultaneously improving nitrogen utilization through changes in intestinal amino acid absorption and microbial nitrogen metabolism.

Multiple studies have confirmed that gastrointestinal flora is closely related to animal health [47]. The correlation analysis provides preliminary evidence linking specific rumen bacteria with host growth and carcass traits. *Prevotella* and *CF231* were positively correlated with longissimus muscle area, whereas *Succiniclasticum* was negatively correlated with pre-slaughter body weight. DMI and dressing percentage were also negatively associated with *CF231*. These relationships suggest that specific microbial taxa may be involved in the metabolic processes connecting rumen fermentation with host nutrient deposition. Taken together, the present findings indicate that reducing dietary CP from 13.4% to 11.0% did not compromise growth performance, feed efficiency, carcass traits, or the major amino acid characteristics of the longissimus muscle when rumen-protected glycine and/or methionine were supplemented. The response was not simply associated with an increase in total dietary protein availability; rather, it may have resulted from improved post-ruminal amino acid balance and metabolic complementarity between glycine and methionine. At the same time, the stable alpha diversity but altered abundance of several dominant bacterial taxa suggests that the dietary intervention modified specific components of the rumen microbial ecosystem without causing broad community disruption. These findings support the potential of targeted rumen-protected amino acid supplementation as a nutritional strategy for reducing dietary CP while maintaining productive performance in Yudong black goats, with potential implications for improving nitrogen-use efficiency and reducing unnecessary dietary protein inputs.

## 5. Conclusions

In conclusion, the present findings indicate that supplementation with rumen-protected glycine and methionine could counteract the adverse effects induced by low-protein diets. Compared with individual GLY or MET supplementation, combined MAG addition tended to show better comprehensive effects. This study systematically evaluates the efficacy of rumen-protected glycine and methionine as dietary additives and provides a theoretical reference for the application of low-protein feeding strategies in goat production. Nevertheless, further comprehensive investigations are still required to elucidate the underlying regulatory mechanisms and verify the long-term effects.

## Figures and Tables

**Figure 1:**
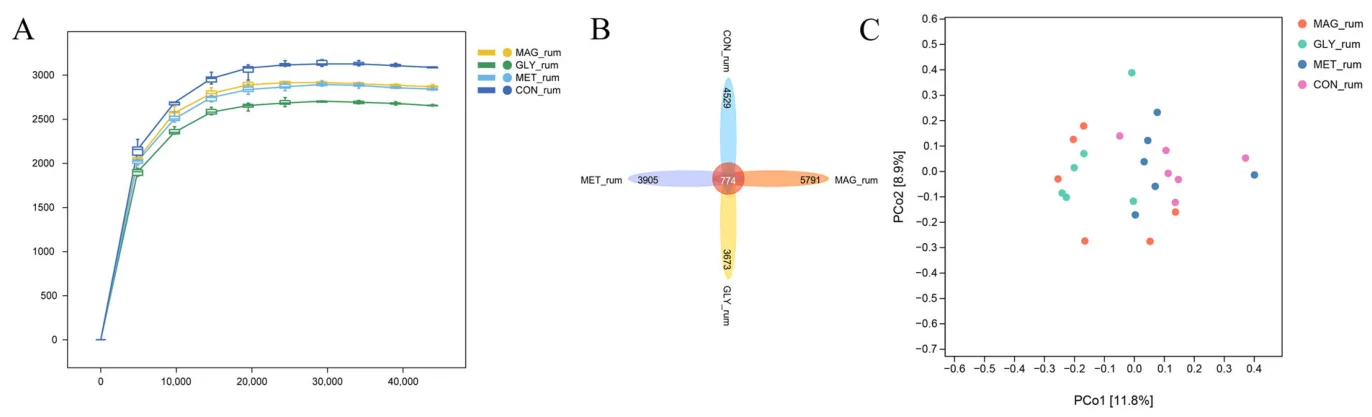
Differential plots of rumen microbial community structure. (**A**) Rarefaction curves of rumen bacteria; (**B**) Venn diagram; (**C**) PCoA plot.

**Figure 2:**
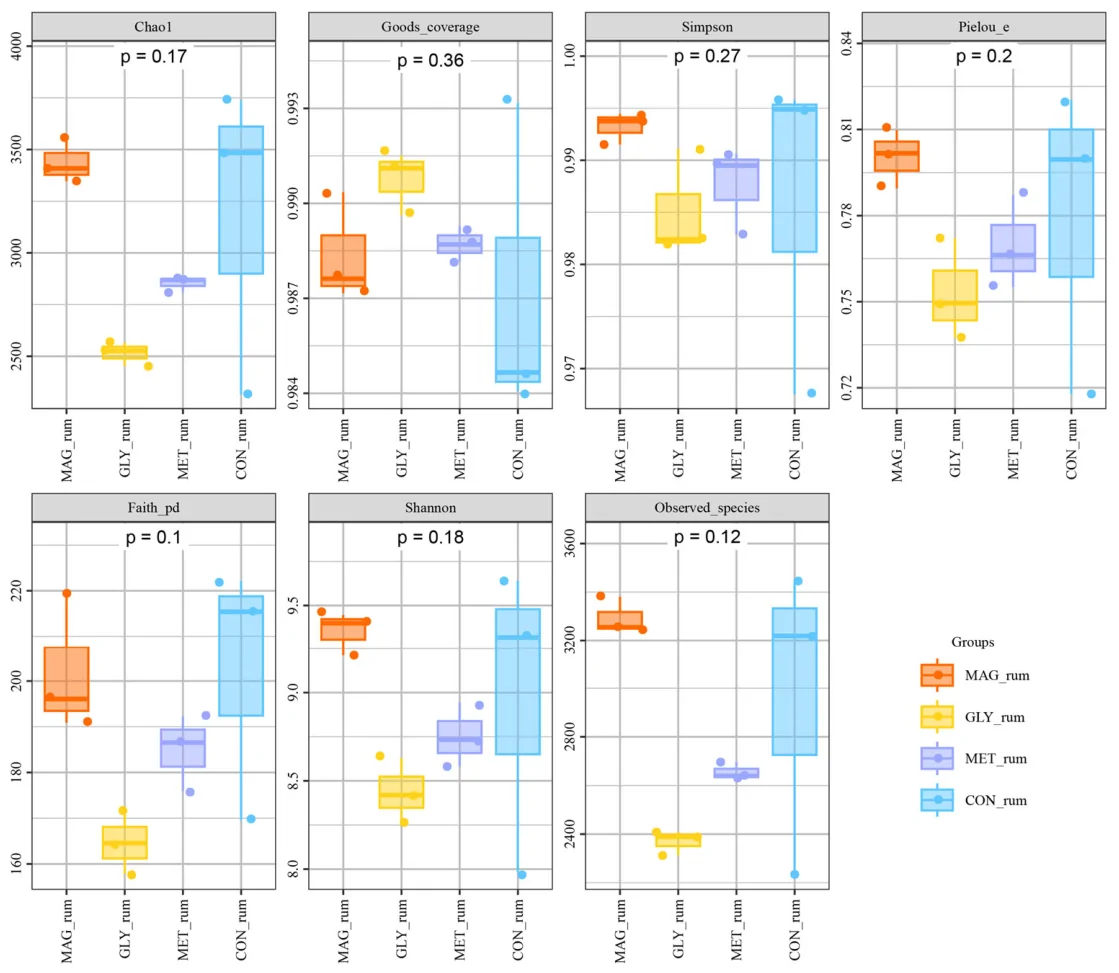
Alpha diversity.

**Figure 3:**
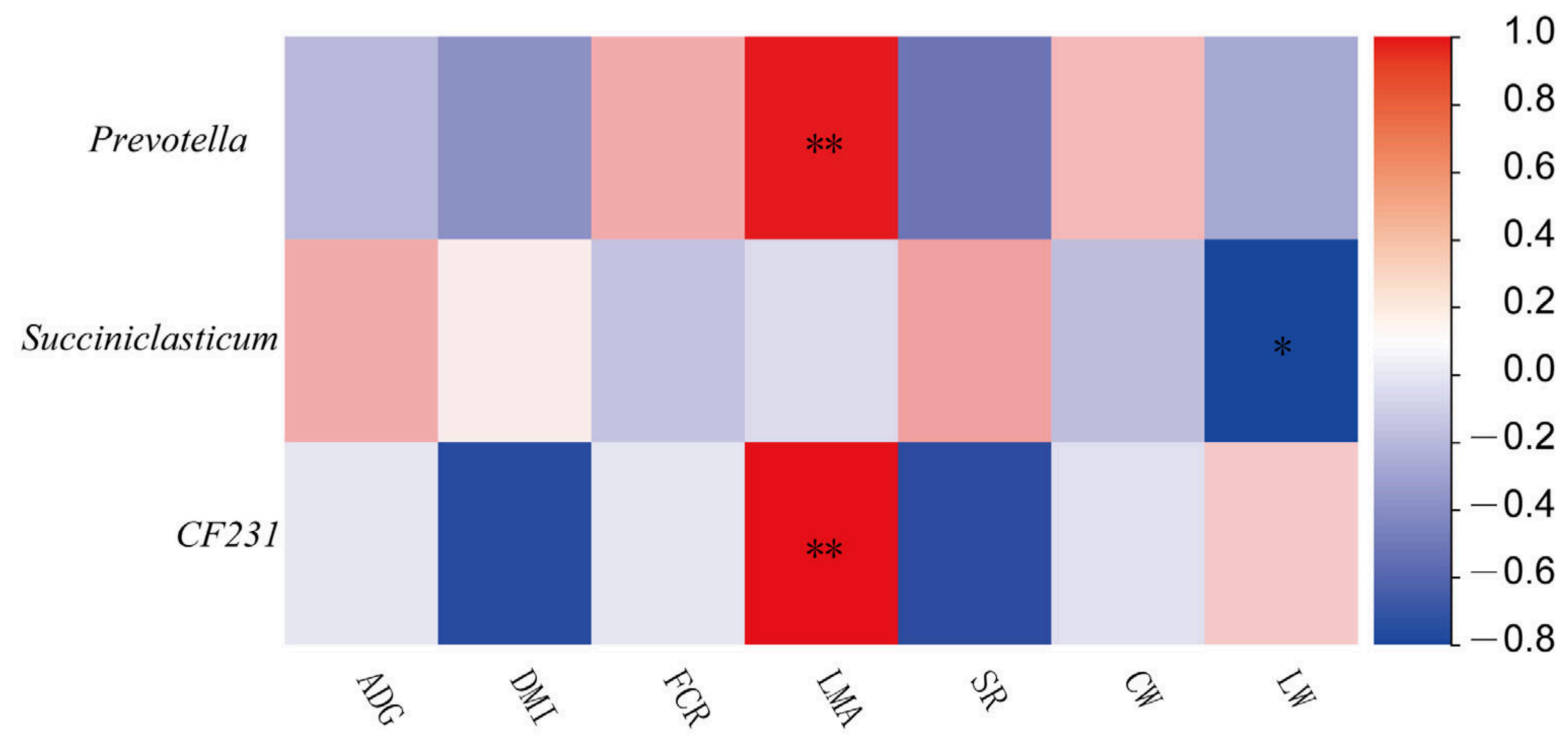
Correlation analysis of dominant bacterial genera with growth performance and slaughter performance. Note: ADG = average daily gain; DMI = dry matter intake; FCR = feed conversion ratio; LMA = longissimus muscle area; SR = slaughter rate; CW = carcass weight; LW = live weight. Colors denote correlation coefficients. Darker red corresponds to a stronger positive correlation, whereas darker blue corresponds to a stronger negative correlation. “*” represents significant correlation, “**” represents extremely significant correlation.

**Figure 4:**
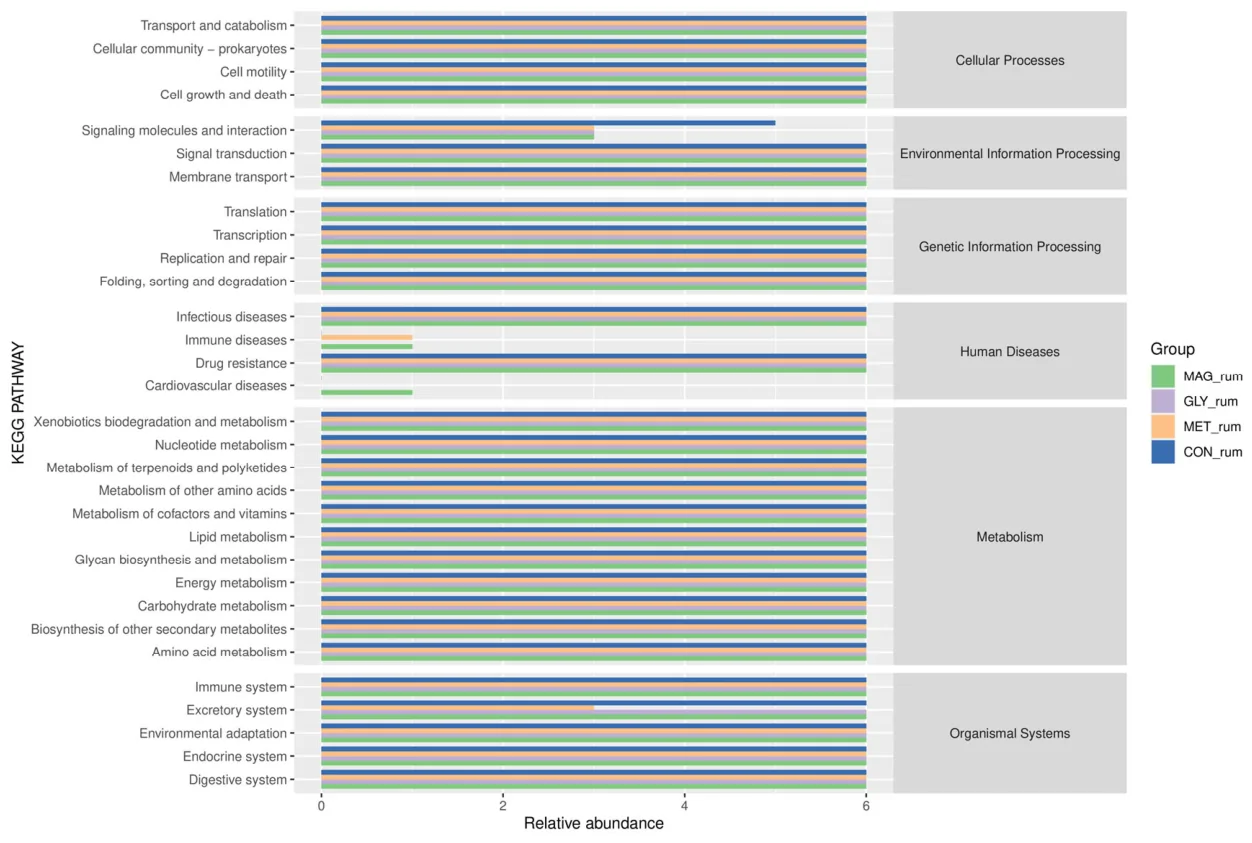
KEGG enrichment analysis.

**Table 1:** Composition and nutritional profile of diet (dry matter basis).

Ingredient	Content (%)		Nutrient Levels	Level	
	CON	Low Protein		CON	Low Protein
Corn	14.70	19.50	Metabolizable energy (ME, MJ/kg)	9.30	9.10
Wheat	3.60	0.90	Crude protein (%)	13.40	11.00
Soybean meal	9.00	3.60	Crude fat (%)	7.90	7.90
Wheat bran	0.90	3.90	Ether extract (%)	2.60	2.60
CaHPO_4_	0.60	0.60	Neutral detergent fiber (%)	22.50	23.80
Vegetable oil	0.60	0.60	Acid detergent fiber (%)	19.00	19.10
CaCO_3_	0.09	0.09	Calcium (%)	0.98	1.02
NaHCO_3_	0.15	0.15	Total phosphorus (%)	0.35	0.37
NaCl	0.15	0.15			
Premix ^1^	0.51	0.51			
Peanut vine	70.00	70.00			
Total	100.00	100.00			

^1^ The premix provides per kg of diet: Copper 12 mg, Zinc 60 mg, Selenium 0.3 mg, Manganese 40 mg, Iron 30 mg, Iodine 0.5 mg, Vitamin A 8000 IU, Vitamin D_3_ 2000 IU, Vitamin E 75 IU, Vitamin B1 10 IU; metabolic energy is a calculated value, while all others are measured values.

**Table 2:** Effects of rumen-protected amino acid supplementation in low-protein diets on the growth performance of Yudong black goats.

Item	CON	GLY	MET	MAG	*p*-Value
IBW, (kg)	21.28 ± 0.32	21.77 ± 0.64	21.19 ± 0.55	21.36 ± 0.55	0.865
FBW, (kg)	29.96 ± 1.00	30.64 ± 1.96	29.71 ± 2.83	29.93 ± 0.63	0.985
ADG, (g/d)	173.75 ± 14.70	177.25 ± 41.58	170.50 ± 46.09	171.25 ± 17.82	0.999
DMI, (g/d)	1337.25 ± 93.47	1211.04 ± 70.58	1232.55 ± 99.50	1166.60 ± 29.51	0.502
FCR	8.78 ± 0.48	8.13 ± 0.85	7.91 ± 0.18	8.27 ± 0.12	0.669

Note: IBW = initial body weight; FBW = final body weight; ADG = average daily gain; DMI = dry matter intake; FCR = feed conversion ratio. Values with no superscript letters indicate no significant difference between groups (*p* > 0.05).

**Table 3:** Effects of rumen-protected amino acid supplementation in low-protein diets on the slaughter performance of Yudong black goats.

Item	CON	GLY	MET	MAG	*p*-Value
Live weight before slaughter/kg	28.99 ± 2.63	28.00 ± 0.66	29.34 ± 2.63	31.41 ± 0.93	0.661
Carcass weight/kg	13.05 ± 1.31	12.88 ± 0.44	13.63 ± 1.62	13.95 ± 0.81	0.899
Dressing percentage/%	44.88 ± 1.08	45.97 ± 0.78	46.15 ± 1.81	44.33 ± 1.26	0.717
Loin eye area/cm^2^	7.81 ± 0.57 ^b^	9.79 ± 0.98 ^ab^	7.99 ± 0.91 ^b^	11.07 ± 0.37 ^a^	0.029

Note: Values with no superscript letters or the same superscript letters indicate no significant difference between groups (*p* > 0.05); in contrast, values with different lowercase superscript letters indicate a statistically significant difference (*p* < 0.05).

**Table 4:** Effects of rumen-protected amino acid supplementation in low-protein diets on serum biochemical parameters and hormone concentrations in Yudong black goats.

Item	CON	GLY	MET	MAG	*p*-Value
Serum biochemical indices					
ALT (U/L)	36.25 ± 1.44	44.00 ± 6.12	42.75 ± 6.14	48.75 ± 3.09	0.344
AST (U/L)	115.50 ± 5.69	138.50 ± 22.78	159.00 ± 36.08	173.75 ± 13.08	0.330
TP (g/L)	66.78 ± 2.21 ^b^	82.63 ± 2.63 ^a^	79.83 ± 4.81 ^a^	75.23 ± 4.02 ^ab^	0.041
ALB (g/L)	39.90 ± 0.30 ^a^	40.43 ± 0.31 ^a^	37.95 ± 0.74 ^b^	37.95 ± 0.76 ^b^	0.017
GLB (g/L)	28.13 ± 1.73	42.20 ± 2.83	41.88 ± 5.55	37.28 ± 4.64	0.091
A/G (%)	1.44 ± 0.09	0.98 ± 0.08	0.96 ± 0.14	1.08 ± 0.17	0.065
ALP (U/L)	387.25 ± 53.54	494.50 ± 150.97	445.00 ± 188.44	582.75 ± 115.17	0.777
GLU (mmol/L)	6.14 ± 0.53	4.97 ± 0.65	5.35 ± 0.39	5.42 ± 0.07	0.384
UREA (mmol/L)	6.90 ± 0.40	9.03 ± 0.95	8.50 ± 0.72	7.90 ± 0.69	0.239
CREA (µmol/L)	49.25 ± 2.72	50.00 ± 7.45	53.50 ± 8.10	51.00 ± 4.51	0.963
CHOL (mmol/L)	1.99 ± 0.27 ^b^	3.02 ± 0.12 ^a^	2.44 ± 0.12 ^ab^	2.20 ± 0.33 ^b^	0.041
TG (mmol/L)	0.27 ± 0.01	0.33 ± 0.03	0.30 ± 0.01	0.30 ± 0.02	0.222
HDL-C (mmol/L)	1.50 ± 0.22	2.01 ± 0.10	1.66 ± 0.12	1.56 ± 0.26	0.271
LDL-C (mmol/L)	0.26 ± 0.02 ^b^	0.51 ± 0.06 ^a^	0.47 ± 0.07 ^a^	0.37 ± 0.03 ^ab^	0.018
NEFA (mmol/L)	1.22 ± 0.16	0.92 ± 0.13	1.20 ± 0.10	0.85 ± 0.08	0.100
Oxidative stress and hormones					
SOD (U/mL)	111.49 ± 2.66	121.90 ± 2.23	109.98 ± 5.39	115.29 ± 7.75	0.381
MDA (nmol/mL)	4.44 ± 0.18	3.70 ± 0.17	4.52 ± 0.37	4.12 ± 0.52	0.353
GSH-PX (U/mL)	1043.94 ± 14.16	1120.65 ± 18.97	1041.33 ± 42.04	1084.96 ± 53.66	0.390
CAT (U/mL)	13.05 ± 0.26	14.16 ± 0.22	12.91 ± 0.49	13.58 ± 0.80	0.320
T-AOC (U/mL)	9.24 ± 0.24	10.03 ± 0.23	9.01 ± 0.49	9.40 ± 0.66	0.431
GH (ng/mL)	1.31 ± 0.32	1.23 ± 0.26	1.06 ± 0.28	1.40 ± 0.09	0.807
G-17 (pmol/L)	0.43 ± 0.09	0.85 ± 0.57	0.22 ± 0.04	2.05 ± 1.83	0.555
IGF-1 (ng/mL)	96.05 ± 17.43	96.88 ± 23.49	120.79 ± 15.99	91.03 ± 4.12	0.610
TNF-α (pg/mL)	23.34 ± 2.44	18.52 ± 4.07	24.05 ± 2.55	23.49 ± 3.33	0.592
IL-4 (pg/mL)	10.99 ± 2.08	11.51 ± 1.28	7.76 ± 0.39	10.31 ± 1.28	0.285
IL-6 (pg/mL)	9.73 ± 0.35	11.06 ± 2.45	14.04 ± 0.90	12.24 ± 1.73	0.305

Note: ALT = alanine aminotransferase; AST = aspartate aminotransferase; TP = total protein; ALB = albumin; GLB = globulin; A/G = albumin–globulin ratio; ALP = alkaline phosphatase; GLU = glucose; UREA = urea; CREA = creatinine; CHOL = cholesterol; TG = triglyceride; HDL-C = high-density lipoprotein-cholesterol; LDL-C = low-density lipoprotein-cholesterol; NEFA = non-esterified fatty acid; SOD = superoxide dismutase; MDA = malondialdehyde; GSH-PX = glutathione peroxidase; CAT = catalase; T-AOC = total antioxidant capacity; GH = growth hormone; G-17 = gastrin-17; IGF-1 = insulin-like growth factor-1; TNF-α = tumor necrosis factor-alpha; IL-4 = interleukin-4; IL-6 = interleukin-6. Values with no superscript letters or the same superscript letters indicate no significant difference between groups (*p* > 0.05); in contrast, values with different lowercase superscript letters indicate a statistically significant difference (*p* < 0.05); significance tendency (0.05 ≤ *p* < 0.10).

**Table 5:** Effects of supplementing rumen-protected amino acids in low-protein diets on amino acids and fatty acids in the longissimus dorsi muscle of Yudong black goats (g/100 g).

Item	CON	GLY	MET	MAG	*p*-Value
Aspartic Acid ^2^	1.55 ± 0.05	1.62 ± 0.08	1.54 ± 0.09	1.62 ± 0.04	0.782
Threonine ^1^	0.82 ± 0.03	0.86 ± 0.04	0.81 ± 0.05	0.86 ± 0.02	0.708
Serine	0.67 ± 0.02	0.68 ± 0.03	0.67 ± 0.04	0.68 ± 0.01	0.978
Glutamic Acid ^2^	2.45 ± 0.09	2.60 ± 0.12	2.49 ± 0.15	2.60 ± 0.06	0.698
Glycine ^2^	0.78 ± 0.01	0.82 ± 0.03	0.79 ± 0.04	0.82 ± 0.02	0.593
Alanine ^2^	1.00 ± 0.03	1.05 ± 0.05	0.99 ± 0.06	1.05 ± 0.02	0.626
Cystine ^1^	0.16 ± 0.00 ^ab^	0.13 ± 0.01 ^c^	0.16 ± 0.01 ^a^	0.13 ± 0.01 ^bc^	0.031
Valine ^1^	0.86 ± 0.03	0.95 ± 0.05	0.84 ± 0.06	0.98 ± 0.02	0.101
Methionine ^1^	0.34 ± 0.02	0.29 ± 0.01	0.32 ± 0.02	0.34 ± 0.04	0.564
Isoleucine ^1^	0.74 ± 0.03	0.82 ± 0.04	0.73 ± 0.05	0.84 ± 0.02	0.080
Leucine ^1^	1.41 ± 0.05	1.48 ± 0.07	1.40 ± 0.08	1.50 ± 0.03	0.598
Tyrosine ^1^	0.43 ± 0.02	0.54 ± 0.06	0.42 ± 0.05	0.58 ± 0.03	0.057
Phenylalanine ^1^	0.70 ± 0.02	0.74 ± 0.04	0.70 ± 0.04	0.75 ± 0.01	0.432
Lysine ^1^	1.58 ± 0.05	1.65 ± 0.08	1.55 ± 0.09	1.66 ± 0.03	0.597
Histidine	0.62 ± 0.03	0.66 ± 0.05	0.62 ± 0.05	0.69 ± 0.03	0.621
Arginine	1.11 ± 0.04	1.15 ± 0.05	1.10 ± 0.06	1.16 ± 0.03	0.804
Proline	0.65 ± 0.02	0.68 ± 0.02	0.65 ± 0.03	0.68 ± 0.02	0.719
C14:0	51.43 ± 13.37	35.00 ± 6.20	34.33 ± 5.07	62.05 ± 3.55	0.083
C15:0	8.05 ± 1.80	6.25 ± 1.16	5.78 ± 0.28	9.48 ± 1.39	0.209
C16:0	827.23 ± 122.93	727.20 ± 139.39	695.03 ± 71.69	953.38 ± 71.74	0.349
C16:1	57.88 ± 11.36	42.37 ± 3.50	50.03 ± 9.41	70.65 ± 8.55	0.180
C17:0	37.80 ± 4.31	42.28 ± 13.48	31.08 ± 0.45	36.15 ± 3.09	0.751
C18:0	709.00 ± 33.78	786.15 ± 182.83	648.95 ± 29.81	804.27 ± 106.65	0.728
C18:1n9c	1918.70 ± 168.58	1944.55 ± 385.02	1763.88 ± 180.61	2131.55 ± 272.39	0.810
C18:2n6c	120.78 ± 7.65	138.83 ± 10.73	128.05 ± 9.69	150.83 ± 7.56	0.152
C18:3n3	4.15 ± 0.33	3.83 ± 0.15	4.43 ± 0.24	4.20 ± 0.10	0.363
C20:1	4.80 ± 0.15	5.20 ± 0.47	5.57 ± 0.23	6.07 ± 0.13	0.064
C20:3n6	5.53 ± 0.12 ^c^	7.93 ± 0.20 ^a^	8.57 ± 0.09 ^a^	6.83 ± 0.34 ^b^	<0.001
C20:4n6	64.63 ± 4.49	75.05 ± 5.03	77.90 ± 5.63	75.80 ± 2.17	0.215
Total AA	15.84 ± 0.55	16.70 ± 0.79	16.10 ± 0.81	16.92 ± 0.37	0.634
EAA ^1^	7.03 ± 0.25	7.45 ± 0.38	7.09 ± 0.36	7.64 ± 0.16	0.446
FAA ^2^	6.71 ± 0.20 ^ab^	7.04 ± 0.18 ^a^	6.32 ± 0.10 ^b^	6.94 ± 0.14 ^a^	0.036

Note: Total AA = total amino acids; ^1^ EAA = essential amino acids; ^2^ FAA = flavor amino acids. Values with no superscript letters or the same superscript letters indicate no significant difference between groups (*p* > 0.05); in contrast, values with different lowercase superscript letters indicate a statistically significant difference (*p* < 0.05); significance tendency (0.05 ≤ *p* < 0.10) and extremely significant difference (*p* < 0.01).

**Table 6:** Taxonomic composition analysis at the phylum level (Top 5).

Items	Groups				
	CON (%)	GLY (%)	MET (%)	MAG (%)	*p*-Value
Firmicutes	0.406 ± 0.030	0.539 ± 0.426	0.422 ± 0.029	0.430 ± 0.047	0.085
Actinobacteria	0.226 ± 0.071	0.302 ± 0.046	0.311 ± 0.062	0.402 ± 0.063	0.275
Bacteroidetes	0.228 ± 0.062 ^a^	0.025 ± 0.011 ^b^	0.146 ± 0.058 ^b^	0.030 ± 0.016 ^b^	0.009
Verrucomicrobia	0.043 ± 0.014	0.047 ± 0.015	0.055 ± 0.015	0.049 ± 0.019	0.966
Proteobacteria	0.042 ± 0.011	0.010 ± 0.002	0.022 ± 0.003	0.030 ± 0.015	0.146

Note: Values with no superscript letters or the same superscript letters indicate no significant difference between groups (*p* > 0.05); in contrast, values with different lowercase superscript letters indicate a statistically significant difference (*p* < 0.05); significance tendency (0.05 ≤ *p* < 0.10).

**Table 7:** Taxonomic composition analysis at the genus level (Top 5).

Items	Groups				
	CON (%)	GLY (%)	MET (%)	MAG (%)	*p*-Value
*Succiniclasticum*	0.070 ± 0.006	0.074 ± 0.010	0.051 ± 0.010	0.078 ± 0.017	0.419
*Oscillospira*	0.015 ± 0.004	0.020 ± 0.009	0.022 ± 0.003	0.007 ± 0.001	0.181
*CF231*	0.007 ± 0.001	0.013 ± 0.001	0.009 ± 0.001	0.013 ± 0.002	0.055
*YRC22*	0.008 ± 0.001	0.007 ± 0.001	0.009 ± 0.003	0.006 ± 0.002	0.679
*Prevotella*	0.005 ± 0.001 ^b^	0.002 ± 0.001 ^b^	0.013 ± 0.004 ^a^	0.003 ± 0.001 ^b^	0.003

Note: Values with no superscript letters or the same superscript letters indicate no significant difference between groups (*p* > 0.05); in contrast, values with different lowercase superscript letters indicate a statistically significant difference (*p* < 0.05); significance tendency (0.05 ≤ *p* < 0.10).

## Data Availability

The original contributions presented in this study are included in the article. Further inquiries can be directed to the corresponding author.
